# Outcomes of the Fontan operation in patients with Ebstein anomaly: An Australia and New Zealand Fontan registry study

**DOI:** 10.1016/j.xjon.2025.06.004

**Published:** 2025-06-12

**Authors:** Michael Daley, Igor E. Konstantinov, Julian Ayer, Ajay Iyengar, David Celermajer, Rachael Cordina, Terry Robertson, Aditya Patukale, Nelson Alphonso, Yves d’Udekem

**Affiliations:** a Department of Cardiac Surgery, Royal Children's Hospital, Melbourne, Victoria, Australia; b Department of Paediatrics, University of Melbourne, Melbourne, Victoria, Australia; c Heart Research Group, Murdoch Children's Research Institute, Melbourne, Victoria, Australia; d The Sydney Children's Hospital Network, Sydney, New South Wales, Australia; e Sydney Medical School, University of Sydney, Sydney, New South Wales, Australia; f Green Lane Paediatric and Congenital Cardiac Service, Starship Children's Hospital, Auckland, New Zealand; g Department of Surgery, Auckland University, Auckland, New Zealand; h Department of Cardiology, Royal Prince Alfred Hospital, Sydney, New South Wales, Australia; i Department of Cardiology, Women's and Children's Hospital, Adelaide, South Australia, Australia; j Department of Cardiac Surgery, Queensland Children's Hospital, Brisbane, Queensland, Australia; k Division of Cardiovascular Surgery, Children's National Heart Institute, Children's National Hospital, Washington, DC

**Keywords:** Fontan, Ebstein anomaly, single ventricle, tricuspid valve, congenitally corrected transposition of the great arteries, outcomes

## Abstract

**Objectives:**

We sought to review the outcomes of patients with Ebstein anomaly (EA) after the Fontan operation.

**Methods:**

Patients with EA were identified from a large binational registry about the Fontan operation. Data were collected from hospital records, registry data, and clinical correspondence.

**Results:**

Of the 1601 patients who underwent a contemporary Fontan operation from 1991 to 2023, 34 patients had EA. Seven patients (21%) had concomitant congenitally corrected transposition of great arteries. Prior Starnes procedure was performed in 18 (53%) patients. Survival after Fontan operation in patients with EA was 92% (95% CI, 70%-98%) and freedom from Fontan failure was 80% (95% CI, 53%-92%) at 10 years. Patients with EA had worse long-term survival (*P =* .01) after Fontan operation and lower freedom from Fontan failure (*P =* .004) compared with other patients with left-ventricle dominance. Patients with EA, who underwent prior Starnes procedure, had 100% survival and freedom from Fontan failure, albeit at a shorter follow-up (median, 4.2 years; range, 13 days-17.7 years), with no difference between patients with prior Starnes and patients with tricuspid atresia (*P* = .76 and *P* = .69, respectively), although comparison was hindered by low numbers. Of the 7 patients with congenitally corrected transposition of great arteries and EA, there were no mortalities; however, 2 patients had Fontan failure at 7.0 and 9.8 years post-Fontan.

**Conclusions:**

Patients with EA have worse long-term outcomes after the Fontan operation compared with other patients with left ventricular dominance. Patients with a prior Starnes procedure appear to have good post-Fontan outcomes, although bias may occur due to small numbers.

**Figure:**
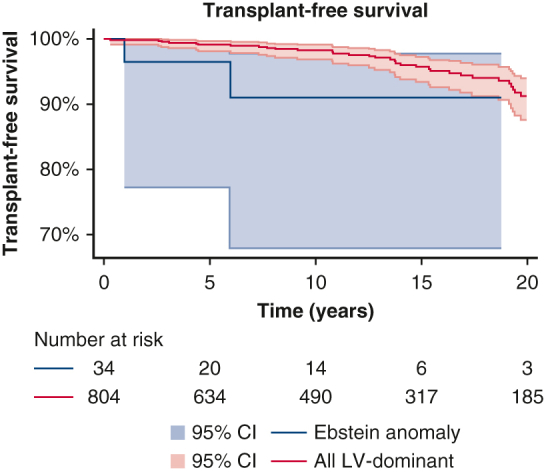
Survival comparison post-Fontan of Ebstein anomaly and other left-dominant morphologies.

**Central Message**

Fontan patients with Ebstein anomaly appear to have worse long-term outcomes than other left-dominant morphologies, but similar to right-dominant morphologies.

**Perspective**

Patients with severe Ebstein anomaly may occasionally necessitate single-ventricle management, the outcomes for which are poorly described. This study demonstrates that patients with Ebstein anomaly appear to have worse outcomes than other left-dominant morphologies. This disparity may be mitigated by the Starnes procedure.

Ebstein anomaly (EA) is a rare congenital cardiac abnormality accounting for <1% of all congenital cardiac defects.^1^ Originally described in 1866, the anomaly consists of a dysplastic tricuspid valve with failure of posterior and septal leaflet delamination, and apical displacement of the annulus resulting in a dilated atrialized portion of the right ventricle, and dysfunctional interventricular dynamics.^2^^,^^3^

The clinical presentation of EA can vary from mild valvular dysmorphia with minimal clinical influence to severe functional pulmonary atresia, circular shunt, and/or refractory cardiogenic shock. Additionally, severe EA is often accompanied by concurrent cardiac anomalies, further clouding ideal management strategies and outcomes. We sought to review the outcomes of Fontan patients with EA in Australia and New Zealand.

## Methods

### Study Population

The study population included patients from the Australian and New Zealand Fontan Registry, a binational registry with ongoing institutional ethics approval and research support (The Royal Children's Hospital Melbourne Human Research Ethics Committee, HREC 36260; February 15, 2012). The institutional review board or equivalent ethics committee of the participating institutions approved the study protocol and publication of data. The patients provided informed written consent for the publication of the study data. Informed written consent is attained on an opt-out basis at the time of the Fontan procedure. The design of the Australian and New Zealand Fontan Registry has been previously described by Iyengar and colleagues.^4^ Of note, the registry includes patients discharged from hospital with an intact Fontan circulation, thus does not include pre-Fontan patients, nor patients with early/operative mortality or predischarge Fontan takedown. Data were collected from hospital records, registry data, and clinical correspondence. Patients with EA were identified from registry coding data, derived from pre-Fontan imaging and operation reports.

### Exclusions/Inclusions

Due to its historical nature, all patients who underwent an atriopulmonary Fontan were excluded from the study. The study period included patients who underwent a Fontan operation from 1991 to 2023.

### Definitions

Fontan failure is defined as the incidence of death, heart transplantation, New York Heart Association functional class III or IV symptoms, plastic bronchitis, protein-losing enteropathy, or Fontan takedown.

### Statistical Analysis

Statistical analyses were performed using Stata version 14 (StataCorp). Continuous variables are reported as mean ± standard deviation if normally distributed, or median (interquartile range and range) if nonnormally distributed. Categorical variables are reported as counts and percentages. Categorical variables are compared using the χ^2^ test. Normally distributed continuous variables are compared using the Student *t* test, and nonparametric continuous variables compared using the Kruskal-Wallis test. Transplant-free survival and freedom from Fontan failure are measured from the date of Fontan operation and estimated using the Kaplan-Meier method. Kaplan-Meier curves are compared using the log-rank test.

## Results

Of the 1841 patients with Fontan circulation, 240 patients with historical atriopulmonary Fontan connection patients were identified and excluded. Of the remaining 1601 patients, 34 patients (2.1%) were identified as having EA. Eighteen patients (53%) were male and no patients had heterotaxy syndrome. Patient demographics are described in Table 1. Concurrent cardiac abnormalities for patients with EA are reported in Table 2, and concomitant procedures at the time of Fontan operation are listed in Table 3.

**Table 1:** Demographic characteristics of patients with Ebstein anomaly (EA)

Characteristic	EA (n = 34)	Other left ventricle dominance (n = 804)	*P* Value
Female sex	16 (47)	332 (41)	.50
Age at Fontan (y)	5.3 ± 2.8	5.4 ± 3.5	.89
Heterotaxy syndrome	0 (0)	28 (3)	.27
Prior Starnes procedure	17 (50)	0 (0)	
Pre-Fontan pulmonary artery pressure (mm Hg)	10.5 ± 2.9	11.3 ± 4.4	.32
Pre-Fontan systemic oxygen saturation (%)	83 ± 6	83 ± 7	.42
Fontan type			.13
LT	4 (12)	183 (23)	
ECC	30 (88)	621 (77)	
Fontan fenestration	16 (47)	294 (37)	.241
Fontan cardiopulmonary bypass time (min)	111 (55-314)	103 (42-311)	.54
Fontan aortic crossclamp time (min)	25 (0-171)	22 (0-147)	.31

Values are presented as n (%), mean ± standard deviation; or median (range).

*LT*, Lateral tunnel; *ECC*, extracardiac conduit.

**Table 2:** Concomitant cardiovascular anomalies for patients with Ebstein anomaly

Concomitant anomalies	Result
Anatomical pulmonary atresia	12 (35)
Functional pulmonary atresia	4 (11)
Pulmonary stenosis	3 (9)
ccTGA	7 (21)
Hypoplastic aortic arch/coarctation	4 (11)
TGA	2 (7)
LVOTO	1 (3)

Values are presented as n (%).

*ccTGA*, Congenitally corrected transposition of the great arteries; *TGA*, transposition of the great arteries; *LVOTO*, left ventricular outflow tract.

**Table 3:** Concomitant procedures at the time of Fontan operation (n = 9)

Concomitant procedure	n
Pulmonary arterioplasty	5
Mitral valve repair	1
Atrial septectomy	1
Clipping of collaterals	1
Stent dilation	1

Three patients died during follow-up at 1, 6, and 25 years post-Fontan, and 5 patients experienced Fontan failure-defining conditions at 1, 6, 7, 10, and 25 years post-Fontan. Long-term survival and freedom from Fontan failure for patients with EA was 92% (95% CI, 70%-98%) and 80% (95% CI, 53%-92%) at 10 years. No patients underwent heart transplantation during the follow-up period.

One patient underwent tricuspid valve repair before their Fontan procedure. This patient required mobilization of their anterior tricuspid leaflet, septal homograft leaflet augmentation, and atrialized right ventricle plication at 8 months of age. Additionally, the patient underwent a Glenn procedure at this time. The tricuspid valve function deteriorated over 13 years and the patient underwent the Fontan procedure at age 14 years. The patient remains well without Fontan failure at 5.9 years post-Fontan follow-up.

Ten patients required further intervention post-Fontan procedure; 3 patients required multiple reinterventions. Three patients (9%) underwent pacemaker implantation and 1 patient (3%) underwent implantable cardiac defibrillator implantation post-Fontan procedure. Reintervention details are described in Table 4. Patients with EA (n = 34) versus other patients with left ventricular dominance (n = 804).

**Table 4:** Reinterventions post-Fontan in patients with Ebstein anomaly (n = 11)

Reintervention	n
Fenestration closure	7
Pacemaker implantation	3
Coiling of systemic-pulmonary collaterals	2
Right ventricle to pulmonary artery conduit	1
Fenestration creation	1
Left pulmonary artery balloon angioplasty	1
Diaphragm plication	1

**Figure 1:**
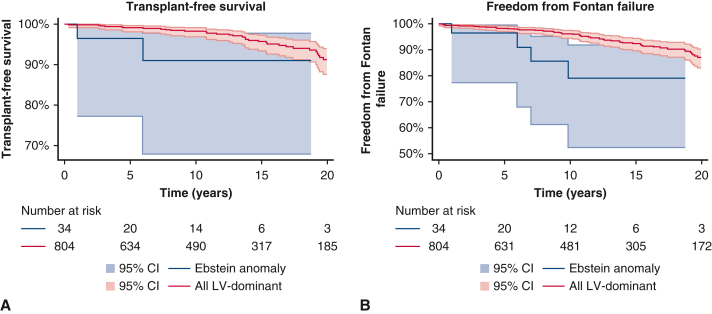
Survival (A) and freedom from Fontan failure (B) of patients with Ebstein anomaly patients compared with other patients with left-ventricular dominance. *P* = .02 and *P* = .005, respectively. 95% CI.

Because right ventricular dominance is known to carry worse long-term outcomes after Fontan operation, comparison was made between patients with EA and other patients with left ventricular dominance. Comparison of baseline demographics is demonstrated in Table 1. Survival and freedom from Fontan failure for other patients with left ventricular dominance was 96% (95% CI, 95% to 97%) and 92% (95% CI, 90%-93%) at 10 years, respectively. Patients with EA had significantly worse outcomes (Figure 1) than other patients with left ventricular dominance (*P* = .02 and *P* = .005, respectively). Cox regression analysis demonstrated hazard ratio [HR] of 3.7 (*P* = .03) for death or transplantation, and HR of 3.6 (*P* = .009) for Fontan failure for patients with EA. This difference in transplant-free survival remained when patients with congenitally corrected transposition of the great arteries (ccTGA) and EA were excluded (HR, 4.6; *P* = .006), although no difference was noted in the incidence of Fontan failure (HR, 2.5; *P* = .12).

Right ventricle-dominant patients had survival and freedom from Fontan failure of 93% (95% CI, 91%-96%) and 84% (95% CI, 81%-88%) at 10 years, respectively. Of note, no difference was demonstrated on comparison between patients with EA and those with right ventricular dominance for transplant-free survival or freedom from Fontan failure (*P* = .63 and *P* = .82, respectively).

### Patients After Starnes Procedure

**Figure 2:**
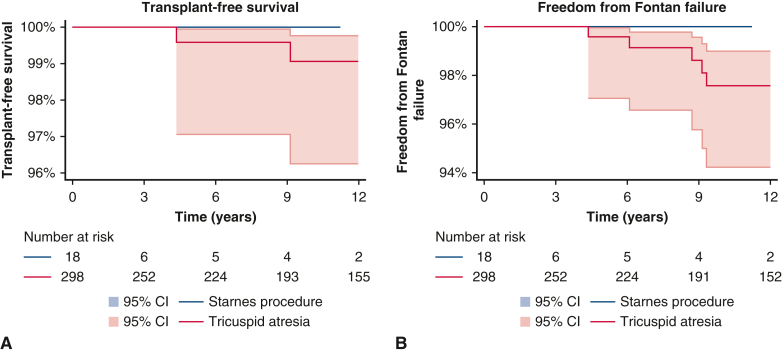
Survival (A) and freedom from Fontan failure (B) in patients after Starnes procedure compared with those with tricuspid atresia. *P* = .76 and *P* = .69, respectively. 95% CI.

Starnes procedure was performed in 18 patients. Median age at the time of Starnes procedure was 7 days (range, 1 day-2.2 years). Median post-Fontan follow-up for patients who had undergone a Starnes procedure was 4.1 years (interquartile range, 1.2-9.1 years), 4 patients of which had follow-up over 10 years post-Fontan. No patients who underwent a Starnes procedure died or developed Fontan failure during the follow-up period. Additionally, no patients underwent attempted tricuspid repair after Starnes procedure. There was no difference in survival or freedom from Fontan failure between patients who underwent Starnes procedure and patients with tricuspid atresia (*P* = .76 and *P* = .69, respectively) (Figure 2).

### Congenitally corrected transposition of the great arteries

Seven patients were identified with ccTGA and EA (21% of patients with EA; 7 out of 34). Two patients underwent prior Starnes procedure at age 22 days and 2.3 years. There were no mortalities during follow-up and 2 patients had Fontan failure at 7.0 and 9.8 years post-Fontan. Late pacemaker implantation was performed in 2 patients (29%) at 6.3 and 9.5 years post-Fontan.

## Discussion

Since first reported, EA has been noted to produce an array of clinical manifestations depending on the severity of valvular and annular dysmorphia, and concurrent anomalies.^3^^,^^5^^,^^6^ Management of patients may vary from conservative management for mild dysfunction to systemic to pulmonary shunts and single-ventricle palliation.^7^ Biventricular repair with the cone technique is the favored approach for patients with repairable valves, and has demonstrated durability when performed in experienced hands.^8^, ^9^, ^10^ However, neonates presenting in extremis with circular shunt or functional pulmonary atresia are often not afforded the luxury of complex valve repair strategies and are managed according to their critical physiology. Our study reports on the mid-to long-term outcomes of patients with EA who have proceeded to Fontan operation. Overall, we demonstrated that Fontan patients with EA experience 92% survival and 80% freedom from Fontan failure at 10 years. These results, although not terrible, leave much room for improvement.

For neonates with severe cyanosis and ductal-dependent circulation, the Starnes procedure has offered a reproducible alternative to high-risk valve repair.^11^, ^12^, ^13^, ^14^ Exclusion of the right ventricle with a fenestrated patch allows for improvement in septal position and left ventricular function, whilst providing pulmonary flow through a modified systemic-to-pulmonary shunt. It is possible that addressing this interventricular physiology early, may improve the systemic ventricle development and lead to better long-term outcomes post-Fontan.

Our study demonstrated worse long-term outcomes for EA patients when compared with other left-ventricular dominant physiologies, with worse survival and a trend toward more Fontan failure. Although the specific reason for this can only be hypothesized, it is possible the altered morphology of the right ventricle and tricuspid annular plane carries a lasting influence on the systemic ventricular function. The Starnes procedure may help to mitigate this physiology, as demonstrated by our excellent post-Fontan outcomes for Starnes patients, albeit with low patient numbers.

The concept of Starnes repair followed by takedown, tricuspid valve repair, and conversion to biventricular physiology has recently gained popularity.^15^^,^^16^ Despite these reports, following Starnes procedure the majority of patients will continue with single-ventricle physiology. Kumar and colleagues^17^ reported the single-ventricle outcomes for patients with EA following Starnes procedure, describing 20 patients who progressed to the Fontan operation. The authors reported 95% and 89% survival at 5 and 10 years’ follow-up, respectively, for patients successfully discharged following a Starnes procedure. Our study reports similar survival of 92% at 10 years post-Fontan procedure for all patients with EA, and 100% survival for Fontan patients who had previous Starnes procedure at a median follow-up of 4.2 years. These excellent Fontan outcomes following the Starnes procedure should encourage clinicians to consider its more liberal use in severe EA, without necessarily committing to a long-term single-ventricle pathway.

Ebsteinoid morphology of the left-sided tricuspid valve in ccTGA is a unique association that has been recognized since 1963, and differs anatomically from its classic right counterpart.^18^^,^^19^ Anderson and colleagues^20^ report a frequently cleft anterior leaflet, hypoplastic morphologic right ventricle, and altered annular plane and subvavular apparatus. These aberrations, and other associated anomalies, suggest a different repair approach may be required for ccTGA with Ebsteinoid valves.^21^, ^22^, ^23^ However, our limited cohort of Fontan patients with ccTGA and recognized Ebsteinoid valves demonstrated acceptable long-term outcomes, as well as the applicability of the Starnes procedure in these patients, if required. The pacemaker requirement for these patients was also substantial (28% [2 out of 7]), demonstrating the higher risk of heart block with these abnormalities.

### Limitations

Our study is limited by its retrospective nature. Similarly, the cohort captured by this study encompasses only patients who survived to Fontan discharge and does not have capacity to examine pre-Fontan data. Follow-up for patients who underwent a Starnes procedure was shorter than other patients, and there is a potential this smaller cohort of patients will experience the expected gradual onset of Fontan failure. Because each patient may have been considered for a biventricular approach, each case was assessed individually at the treating institution with consensus decision to pursue single-ventricle palliation.

## Conclusions

Our study demonstrates that patients with EA have worse long-term outcomes after the Fontan operation compared with other patients with left-ventricular dominance. Although limited by low numbers, it appears the Starnes procedure may mitigate this risk in some patients, with good midterm outcomes. Patients with ccTGA present a unique anatomical challenge that warrants further investigation. Further investigation into the pre-Fontan management of patients with severe EA, and long-term effects of the Starnes procedure are warranted.

## Conflict of Interest Statement

The authors reported no conflicts of interest.

The *Journal* policy requires editors and reviewers to disclose conflicts of interest and to decline handling or reviewing manuscripts for which they may have a conflict of interest. The editors and reviewers of this article have no conflicts of interest.
